# Characterization of the white matter networks in schizophrenia patients with metabolic syndrome undergoing risperidone or clozapine treatment

**DOI:** 10.3389/fnins.2025.1579810

**Published:** 2025-04-02

**Authors:** Xinyan Wu, Xinyue Chen, Kaike Liao, Rui Yu, Yuwei Chen, Kang Li, Nian Liu

**Affiliations:** ^1^Department of Radiology, Affiliated Hospital of North Sichuan Medical College, Nanchong, China; ^2^Chongqing General Hospital, Chongqing University, Chongqing, China

**Keywords:** schizophrenia, metabolic syndrome, white matter structural networks, risperidone, clozapine

## Abstract

**Background:**

The characteristics of the white matter network in schizophrenia patients with metabolic syndrome (MetS) remain unclear. This study analyzed white matter network characteristics in schizophrenia patients with MetS undergoing risperidone or clozapine treatment and explored their potential association with metabolic index and cognitive function.

**Methods:**

Diffusion tensor imaging was used to evaluate 19 schizophrenia patients with comorbid MetS (MetS-SZ) and 20 schizophrenia patients without MetS (nMetS-SZ), as well as 25 healthy controls (HC). Differences in these network metrics were compared among these through groups using ANCOVAs and post-hoc testing. Associations between differential network metrics and clinical characteristics were also analyzed.

**Results:**

Relative to HC individuals, both MetS-SZ and nMetS-SZ patients exhibited a reduction in bilateral thalamic degree centrality (DC) and nodal efficiency (NE). Relative to the HC group, MetS-SZ patients exhibited reductions in both global efficiency and local efficiency, lower levels of DC in the superior occipital gyrus, and reduced NE in the prefrontal and occipital cortices. Relative to nMetS-SZ patients, MetS-SZ patients also exhibited reduced global efficiency and local efficiency, together with decreases in NE in the prefrontal cortex, medial and paracentral cingulate gyrus, occipital cortex, angular gyrus, and temporal pole. Impairments in executive function were associated with reduced NE values in the right angular gyrus, left medial and paracingulate gyrus. Increases in waist circumference and hip circumference, as well as impairments in executive function, were associated with reductions in NE among patients with schizophrenia.

**Conclusion:**

Specific changes in the structure of the white matter network accompanying cognitive deficits were observed in MetS-SZ patients. These results offer new insight into the mechanisms underlying the neural network in schizophrenia patients with MetS.

## 1 Introduction

Schizophrenia is a psychiatric disorder that can present with an array of cognitive deficits together with both negative and positive symptoms (Jauhar et al., 2022). The median lifetime prevalence of this disorder is approximately 4.0 per 1,000 persons, and affected individuals face mortality rates 2–3 times those of healthy subjects (Pillinger et al., 2019; Yoca et al., 2020). The most commonly used medications to manage schizophrenia are antipsychotics, which can significantly alleviate psychotic symptoms (Lähteenvuo and Tiihonen, 2021), with two of the most widely used antipsychotics being risperidone and clozapine. The prolonged use of these drugs, however, can contribute to elevated rates of metabolic syndrome (MetS) among schizophrenia patients (Ballon et al., 2014; De Hert et al., 2011; De Hert et al., 2009; Zhang et al., 2021). Indeed, an estimated 9–24% of patients with this disorder undergoing risperidone treatment are affected by MetS (Saddichha et al., 2008), while upwards of 40% of patients treated with clozapine suffer from MetS (Quek et al., 2022). MetS is a condition that entails a series of metabolic abnormalities including hypertension, hyperglycemia, dyslipidemia, abdominal obesity, and type 2 diabetes mellitus (Fahed et al., 2022; Hasnain et al., 2009). MetS are associated with a higher risk of cardiovascular disease and can lead to an increased risk of death in patients, which in turn reduces patient adherence (Raedler, 2010). As a consequence, efforts to clarify the mechanistic basis for MetS in schizophrenia patients are vital to improve patient adherence and minimize the discontinuation of these medications.

In previous studies, the molecular etiology of MetS in schizophrenia patients undergoing risperidone and clozapine treatment has been explored at the biochemical level (Molina et al., 2021; Zeng et al., 2021), whereas few comparable neuroimaging analyses have been conducted. To date, only two studies (de Nijs et al., 2018; Zhou et al., 2023) published to date have examined the brain networks associated with comorbid schizophrenia and MetS. One study on gray matter revealed that the gray matter volume in the reward circuit region was reduced in schizophrenia patients with MetS (de Nijs et al., 2018), while a separate fMRI study demonstrated alterations in the functional connectivity of the cerebellar cognitive module, bilateral posterior precuneus, and left middle frontal gyrus in individuals with both disorders (Zhou et al., 2023). Obesity has also been studied in this context given that is a risk factor for MetS development. In one obesity-focused white matter study, a reduction in white matter fiber structural integrity was noted in obese schizophrenia patients following antipsychotic treatment (Spangaro et al., 2018). White matter studies can provide valuable insight into the anatomical connections in the brain, and studies of these white matter networks may reveal the interconnected interactions among different regions of the brain, highlighting the complex nature of brain functionality. Whether the white matter network of schizophrenia patients with MetS exhibits any characteristic changes, however, remains to be determined.

Diffusion tensor imaging (DTI) can afford indirect measurements of white matter organization at the microstructural level (Jones, 2008), but it cannot provide insight into the structure or function of the white matter network. Indeed, efforts to examine the formation of functional networks from white matter connections are not possible using current technologies. Graph theory approaches, which have been used to study neural mechanisms in psychiatric disease (Bassett et al., 2018; Braun et al., 2018), enable the topological characterization of brain networks, with metrics like global efficiency (network integration), local efficiency (local sub-network processing), degree centrality (node significance by direct connections), and node efficiency (role in information flow based on connectivity) helping to assess the network’s overall and local information processing efficiency. The combination of DTI and graph theory can thus support efforts to more fully understand the structural and functional features of brain white matter networks in schizophrenia patients undergoing risperidone or clozapine treatment.

This study was designed to the goal of using DTI and graph theory to explore the metrics that characterize the white matter in schizophrenia patients with MetS undergoing risperidone or clozapine treatment. Correlations between distinct brain regions, metabolic indices, and cognitive function were also examined. We hypothesized that patients with comorbid MetS have characteristic alterations in white matter network that may be associated with cognitive functions.

## 2 Materials and methods

The study protocol was approved by the Research Ethics Committee of the Affiliated Hospital of Sichuan North Medical College (2022ER504-1), and all participants or their legal guardian provided written informed consent.

### 2.1 Participants

In this study, GPower3.1.9 software was used to calculate the required sample size, and the effect size was set to 0.5 and the significance level α was set to 0.05. The calculation results showed that 14 subjects in each group needed to be included in order to achieve the statistical testing power of 0.8, totaling 42 subjects. This study enrolled 43 patients with schizophrenia (illness duration range: 2–25 years) and 25 age-, sex-, and education-matched healthy controls (HCs). Patients with schizophrenia were enrolled from Wusheng Psychiatric Hospital and Bashu Psychiatric Hospital, and were separated based on their MetS status into 20 patients with comorbid MetS (MetS-SZ group) and 23 without comorbid MetS (nMetS-SZ group). All of these patients had been undergoing treatment in these psychiatric facilities for an extended period where they had received appropriate care. The HCs were local community members who were enrolled after advertisement or by word of mouth. These HCs were confirmed to be free of any lifetime history of psychiatric illness based on the non-patient version of the Structured Clinical Interview for DSM-IV Axis I Disorders (SCID-NP), and they also confirmed that their first-degree relatives did not have any history of psychosis. Metabolism-related data from the last 3-month physical examination of HCs were used to exclude healthy individuals with MetS according to the race-adjusted National Cholesterol Education Program-Third Adult Treatment Panel criteria (Li et al., 2021).

To be eligible for study inclusion, schizophrenia patients had to: (1) have been diagnosed based on the international classification of disorders (ICD-10) (Castagnini and Berrios, 2009); (2) be ≥18 years of age and right-handed; (3) have a schizophrenia duration ≥2 years based on data from patients, their family, medical records, and the Nottingham Morbidity Schedule (Singh et al., 2005); and (4) be undergoing stable risperidone or clozapine treatment.

The criteria used to diagnose MetS among schizophrenia patients included race-specific adjustments (waist circumference) based on the criteria of the International Cholesterol Education Programme-Third Adult Treatment Panel (Li et al., 2021). Briefly, patients were diagnosed with MetS if they exhibited at least three of the following: (1) elevated waist circumference (≥90 cm and ≥80 cm for Chinese men and women, respectively); (2) elevated triglycerides (TG) (≥1.7 mmol; 150 mg/dL) or be undergoing medical treatment; (3) High-density lipoprotein (HDL-C) < 1.03 mmol (40 mg/dL) or <1.3 mmol (50 mg/dL) for males and females, respectively; (4) systolic blood pressure (SBP) ≥130 mmHg or diastolic blood pressure (DBP) ≥ 85 mm Hg; (5) fasting plasma glucose (FPG) > 5.6 mmol (100 mg/dL).

Patients were excluded from all groups if they: (1) exhibited any MRI contraindications; (2) had any history of organic mental disorders or head trauma; (3) had experienced serious physical illnesses or neurological disorders; (4) were affected by any metabolic diseases; (5) had any history of substance or drug abuse; (6) were undergoing treatment with antipsychotics other than risperidone or clozapine; or (7) exhibited poor DTI image quality.

Based on the criteria detailed above, 4 patients (1 MetS-SZ, 3 nMetS-SZ) were excluded, while 19 MetS-SZ patients (age: 42.16 ± 8.78 years, M/F: 10/9, disease duration: 9 (5, 11) years), 20 nMetS-SZ patients (age: 35.25 ± 9.19 years, M/F: 15/5, disease duration: 13.15 ± 6.627 years), and 25 HCs (age: 37.84 ± 8.77 years, M/F: 14/11) were retained for the final analyses. None of the enrolled schizophrenia patients had a history of diagnosed MetS prior to treatment, as determined based on clinical records obtained from the psychiatric institutions. Of patients in the MetS-SZ group, 10 were undergoing risperidone monotherapy, 3 were undergoing clozapine monotherapy, and 6 were undergoing combined risperidone and clozapine treatment, while the corresponding numbers in the nMetS-SZ group were 3, 3, and 14, respectively. Antipsychotic drug dosages in this study were converted into chlorpromazine equivalents (Gardner et al., 2010).

### 2.2 Laboratory and clinical examinations

The primary serum metabolic indices analyzed for these schizophrenia patients in the present study included total cholesterol, TG, HDL-C, and LDL-C levels. FPG was determined using an electronic blood glucose meter using blood from the fingertip. SBP and DBP were measured with the patient seated after at least 10 min of rest. Waist circumference was determined at the umbilicus level while standing, and hip circumference was assessed at the gluteal peak level while standing. Measurements were made lightly against the skin. Height and weight were determined with standard instruments. Body mass index (BMI) represented the weight (kg) divided by height squared (m^2^).

The Brief Assessment of Cognition in Schizophrenia scale (BACS) (Keefe et al., 2004) was utilized to evaluate cognition in all participants, as it is a thoroughly validated scale that can assess the working and verbal memory, processing and motor speed, executive function, and verbal fluency of subjects, with lower scale scores corresponding to worse cognitive performance. The Therapeutic Emergency Symptom Scale (TESS) (Zhang et al., 2014) was also administered to schizophrenia patients with and without MetS to assess medication side effects, while extrapyramidal antipsychotic side effects in these patients were analyzed with the Extrapyramidal Side Effects Scale (RSESE) (Simpson and Angus, 1970). Higher TESS and RSESE scale scores are indicative of more pronounced antipsychotic medication-related side effects.

### 2.3 Image acquisition and preprocessing

A 3-T scanner (GE DISCOVERY MR750, WI, United States) with a 32-channel phased array head coil was utilized for all MRI scanning in this study at the Affiliated Hospital of North Sichuan Medical College. Scanning was performed while subjects were in the supine position, using foam pads and ear plugs to stabilize their heads and reduce scanning-related noise. DTI data were collected with a diffusion-weighted planar spin echo EPI sequence (repetition time [TR] = 7,900 ms, echo time [TE] = 86 ms, flip angle [FA] = 90°) with a 104 × 104 matrix over a 208 × 208 mm field of view, collecting 60 axial slices (2 mm thick) covering the entirety of the brain with no gaps. The voxel resolution was 2 mm × 2 mm × 2 mm, providing anisotropic voxel sizes in all three dimensions. The scan includes two *b*-values: one with application of diffusion-sensitizing gradients (*b* = 1,000 s/mm^2^) and one lack of diffusion weighting (*b* = 0) along 30 different directions. The acquisition of structural images using high-resolution T1-weighted parameters involved the use of a scrambled phase gradient echo sequence (TR = 7 ms, TE = 3 ms, FA = 9°) with a 256 × 256 matrix over a 256 × 256 mm field of view with 188 contiguous axial slices (1 mm thick).

Preprocessing of the DTI data was undertaken using FMRIB Software Library (FSL) software.^1^ Initially, the data were converted into the NIFTI format, after which they were corrected for head movement and eddy current distortions through the application of an affine alignment to the b0 image for each diffusion-weighted image. Scalping was used to remove non-brain structures including the scalp and skull from the collected images (Smith, 2002). Images of poor quality were excluded by checking the quality of the imaging data. Diffusion tensors for each voxel were then estimated. Average fiber number (FN) and fractional anisotropy (FA) were additionally computed.

### 2.4 Network construction

White matter network construction was achieved with the PANDA pipeline tool^2^ (Cui et al., 2013). The definitions of network nodes and edges have been detailed in prior studies, and are essential for these analyses (Shu et al., 2009; Gong et al., 2009). Here, individual T1-weighted images were co-registered to b0 images in the DTI space, followed by nonlinear transformation into the ICBM152 T1 template in the Montreal Neurological Institute (MNI) space. Automated anatomical labeling atlases (AAL90 templates) from the MNI space were then converted to the local DTI image space. Ninety cortical and subcortical regions (45 each in the right and left hemispheres) were then established as white matter network nodes.

Network edges were established with a deterministic fiber bundle map, obtaining whole-brain fiber tracking maps with the continuum tracking method from the deterministic tracking algorithm in the open-source PANDA package for MATLAB. This algorithm stops tracking at angles >45° or when reaching a voxel with an FA < 0.2. The impact of false connections was reduced by setting a threshold value, defining structural connections by the presence of at least three fibers between any two nodes. Average fiber number (FN) values were calculated as their weights (Zhu et al., 2020), thereby establishing weighted symmetric anatomical 90 × 90 structural connectivity matrices for all subjects in this study that were used for graph theory-based network analyses.

### 2.5 Network analyses

Global and nodal network metrics for all participants in this study were computed with GRETNA.^3^ The small-world property for subjects in these three groups was fulfilled by calculating the sparsity threshold. Global metrics included small-world properties, characteristic path length, global efficiency, and local efficiency. Small-world properties included normalized clustering coefficients, normalized feature path lengths, and small-world scale. Regional metrics for individual nodes included degree centrality (DC), and node efficiency (NE).

Brain networks are considered to have local information processing and global information integration capacities when γ > 1, λ ≈ 1, and *σ* = γ/λ > 1, and the network is meaningful (Wu et al., 2016; Grabow et al., 2015). Global efficiency serves as a metric for the information integration capacity of the overall network, whereas local efficiency measures the ability of localized networks to process information (Watts and Strogatz, 1998; Bassett and Bullmore, 2017). DC was defined as the number of edges or nodes directly connected to a given node, providing insight into the importance of a particular node (Bullmore and Sporns, 2009). NE, in contrast, corresponds to the probability of a given node being connected to all other nodes in the network, thus reflecting the role that that node plays in transmitting information within the network (Geng et al., 2017). A node is more efficient if it presents with more internodal connections in a given brain network.

### 2.6 Statistical analyses

R software^4^ was used to perform statistical analysis on patient demographic and clinical data. One-way ANOVAs were used to compare age and education levels among these three groups, while sex was analyzed with a chi-square test. Serum metabolic indices and clinical scale scores were compared with two-sample *t*-tests or nonparametric Mann–Whitney U tests (when non-normally distributed).

Analyses of covariance (ANCOVAs) were used to analyze differences in network metrics among groups, controlling for the effects of age, sex, and education. A false discovery rate (FDR) corrected *p* < 0.05 was considered significant when analyzing nodal properties. Fisher’s Least Significant Difference (LSD) test was used for post-hoc comparisons analyzing significant differences between groups in a two-by-two manner.

Correlations between significant brain metrics, serum metabolic indices, and clinical scale scores were evaluated separately for the MetS-SZ group, the MetS-SZ group, the combined MetS-SZ + nMetS-SZ group and HC group, after controlling for age, sex, education, disease duration, and chlorpromazine equivalents. *p* < 0.05 was considered significant.

## 3 Results

### 3.1 Demographic, laboratory, and clinical data

No differences in age, sex, or educational distributions were observed among the HC, MetS-SZ, and nMetS-SZ groups, nor did illness duration or daily antipsychotic dosages (chlorpromazine equivalents) differ significantly between the MetS-SZ and nMetS-SZ groups (all *p* > 0.05), as shown in Table 1.

**Table 1:** Demographic and clinical metabolic indicators of the study participants.

	MetS-SZ (*n* = 19)	nMetS-SZ (*n* = 20)	HC (*n* = 25)	Statistic (ANOVA, *t* or χ^2^)	*p* value	*Post-hoc* analyses		
						MetS-SZ vs. nMetS-SZ	MetS-SZ vs. HC	nMetS-SZ vs. HC
Age, y	42.160 ± 8.780	35.250 ± 9.190	37.840 ± 8.770	1.997	0.163^a^	0.055	0.349	1.000
Sex (M/F)	10/9	15/5	14/11	0.011	0.917^b^	0.480	1.000	0.600
Education, y	7.470 ± 2.440	7.750 ± 2.120	9.200 ± 4.110	3.525	0.065^a^	1.000	0.230	0.380
Illness duration, y	10.110 ± 6.950	13.150 ± 6.627	–	−1.619	0.105^d^	–	–	–
CPZ equivalents (mg)	228.290 ± 127.800	290.630 ± 172.100	–	−1.603	0.112^d^	–	–	–
BMI (kg/m2)	25.640 ± 3.560	21.510 ± 2.430	23.710 ± 3.260	2.526	0.117^a^	<0.001***	0.139	0.065
Systolic blood pressure	129.950 ± 17.700	113.000 ± 15.160	–	3.204	0.003^c^ **	–	–	–
Diastolic blood pressure	84.160 ± 11.820	80.500 ± 9.350	–	−1.055	0.298^d^	–	–	–
Waist measurement	91.260 ± 8.180	80.300 ± 7.380	–	4.335	<0.001^c^ ***	–	–	–
Hip measurement	96.110 ± 6.070	90.250 ± 5.210	–	3.123	0.003^c^ **	–	–	–
TG	2.330 ± 1.200	1.450 ± 0.590	–	−2.838	0.005^d^ **	–	–	–
TC	4.410 ± 0.897	3.860 ± 1.040	–	1.773	0.085^c^	–	–	–
HDL	1.720 ± 0.420	1.850 ± 0.410	–	−0.960	0.344^c^	–	–	–
LDL	3.130 ± 0.612	3.130 ± 0.504	–	0.009	0.992^c^	–	–	–

The data are presented as the means ± standard deviation. “—,” not applicable; TG, triglycerides; TC, total cholesterol; HDL, high-density lipoprotein; LDL, low-density lipoprotein. MetS-SZ, schizophrenia patients with metabolic syndrome; nMetS-SZ, schizophrenia patients without metabolic syndrome; HC, healthy controls.

a One-way ANOVA was employed to examine the age disparity among the three groups.

b χ^2^ test was employed to examine the gender disparity among the three groups.

c Two-sample *t*-test was employed to assess the disparity in normally distributed data between the two schizophrenia groups.

d Mann-Whitney U tests was employed to assess the disparity in non-normally distributed data between the two schizophrenia groups.

*Significant group difference at *p* < 0.05; **p < 0.01; ***p < 0.001; ****p < 0.0001.

Relative to nMetS-SZ patients, those in the MetS-SZ group resented with significantly higher waist circumference (*t* = 4.335, *p* < 0.001), hip circumference (*t* = 3.123, *p* = 0.003), SBP (*t* = 3.204, *p* = 0.003), and TG (*Z* = −2.838, *p* = 0.005), whereas no significant differences in DBP, TC, HDL, BMI or LDL were noted (*p* > 0.05) (Table 1).

Relative to HC individuals, MetS-SZ and nMetS-SZ groups had lower BACS total scores and scores for the verbal memory, working memory, movement speed, verbal fluency, attention, and executive function dimensions (*p* < 0.001). However, there were no differences between the MetS-SZ and nMetS-SZ groups in terms of BACS scores or scores for any of these individual dimensions. TESS and RESRS scale scores similarly showed no significant differences between the MetS-SZ and nMetS-SZ groups (*p* > 0.05), as shown in Table 2.

**Table 2:** Difference of scale scores among MetS, nMetS, and HC groups.

	MetS-SZ (*n* = 19)	nMetS-SZ (*n* = 20)	HC (*n* = 25)	Statistic (ANOVA, *t* or χ^2^)	*p* value	*Post-hoc* analyses		
						MetS-SZ vs. nMetS-SZ	MetS-SZ vs. HC	nMetS-SZ vs. HC
BACS total	128.420 ± 52.950	136.150 ± 42.750	232.880 ± 35.990	45.200	<0.001^a^	1.000	<0.001***	<0.001***
Verbal memory	14.680 ± 12.850	17.000 ± 10.150	37.600 ± 12.140	26.093	<0.001^a^	0.900	<0.001***	<0.001***
Working memory	10.530 ± 8.660	12.600 ± 8.240	26.680 ± 2.580	39.997	<0.001^a^	0.830	<0.001***	<0.001***
Movement speed	64.740 ± 14.330	65.900 ± 14.940	79.120 ± 5.290	14.127	<0.001^a^	1.000	0.001***	0.003**
Verbal fluency	13.530 ± 9.470	15.550 ± 7.100	27.560 ± 7.910	19.553	<0.001^a^	0.830	<0.001***	<0.001***
Attention	16.370 ± 11.960	16.950 ± 10.620	45.840 ± 18.350	25.039	<0.001^a^	1.000	<0.001***	<0.001***
Executive function	8.580 ± 7.250	8.580 ± 7.010	18.080 ± 3.090	27.245	<0.001^a^	1.000	<0.001***	<0.001***
TESS	6.260 ± 5.980	7.500 ± 6.100	–	−0.720	0.480^b^	–	–	–
RSESE	1.110 ± 1.940	1.650 ± 2.910	–	−0.675	0.510^b^	–	–	–

The data are presented as the means ± standard deviation. “—,” not applicable. BACS, Brief Assessment of Cognition in Schizophrenia scale; TESS, Therapeutic Emergency Symptom Scale; RSESE, Extrapyramidal Side Effects Scale. MetS-SZ, schizophrenia patients with metabolic syndrome; nMetS-SZ, schizophrenia patients without metabolic syndrome; HC, healthy controls.

a One-way ANOVA was employed to examine the scale scores among the three groups.

b Mann-Whitney U tests was employed to assess the disparity in non-normally distributed data between the two schizophrenia groups.

*Significant group difference at *p* < 0.05; **p < 0.01; ***p < 0.001; ****p < 0.0001.

### 3.2 Differences in network metrics among groups

#### 3.2.1 Global metrics

ANCOVAs were used to analyze global network connectivity in the three patient groups, revealing significant differences among groups in terms of global efficiency and local efficiency (all *p* < 0.05, FDR corrected), while the characteristic path length, standardized clustering coefficients, standardized shortest path length, and small-world attributes were comparable across groups (*p* > 0.05).

**Figure 1:**
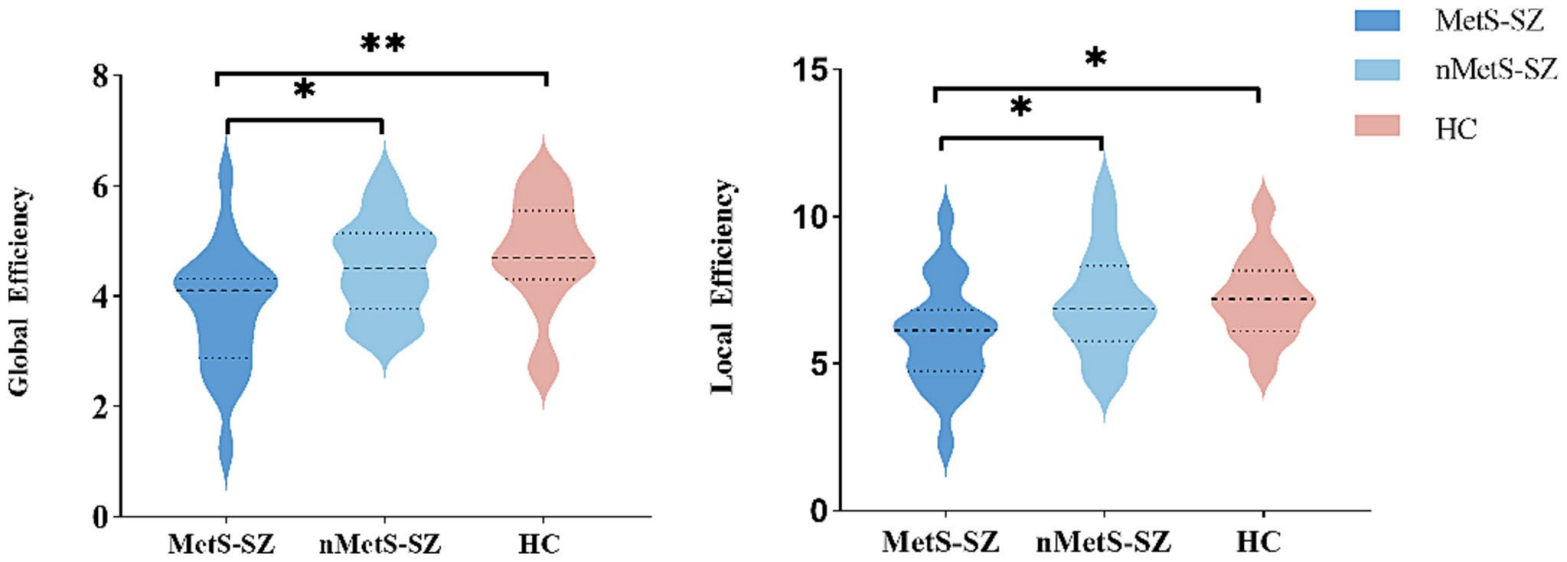
Group differences in global topological properties. MetS-SZ, schizophrenia patients with metabolic syndrome; nMetS-SZ, schizophrenia patients without metabolic syndrome; HC, healthy individuals. An asterisk designates network metrics with significant group differences. *p < 0.05; **p < 0.01.

Relative to HCs, both nMetS-SZ and MetS-SZ patients exhibited marked reductions in global efficiency and local efficiency. Global metrics showed no significant differences between the nMetS-SZ and HC groups, as shown in Figure 1.

#### 3.2.2 Nodal metrics

**Figure 2:**
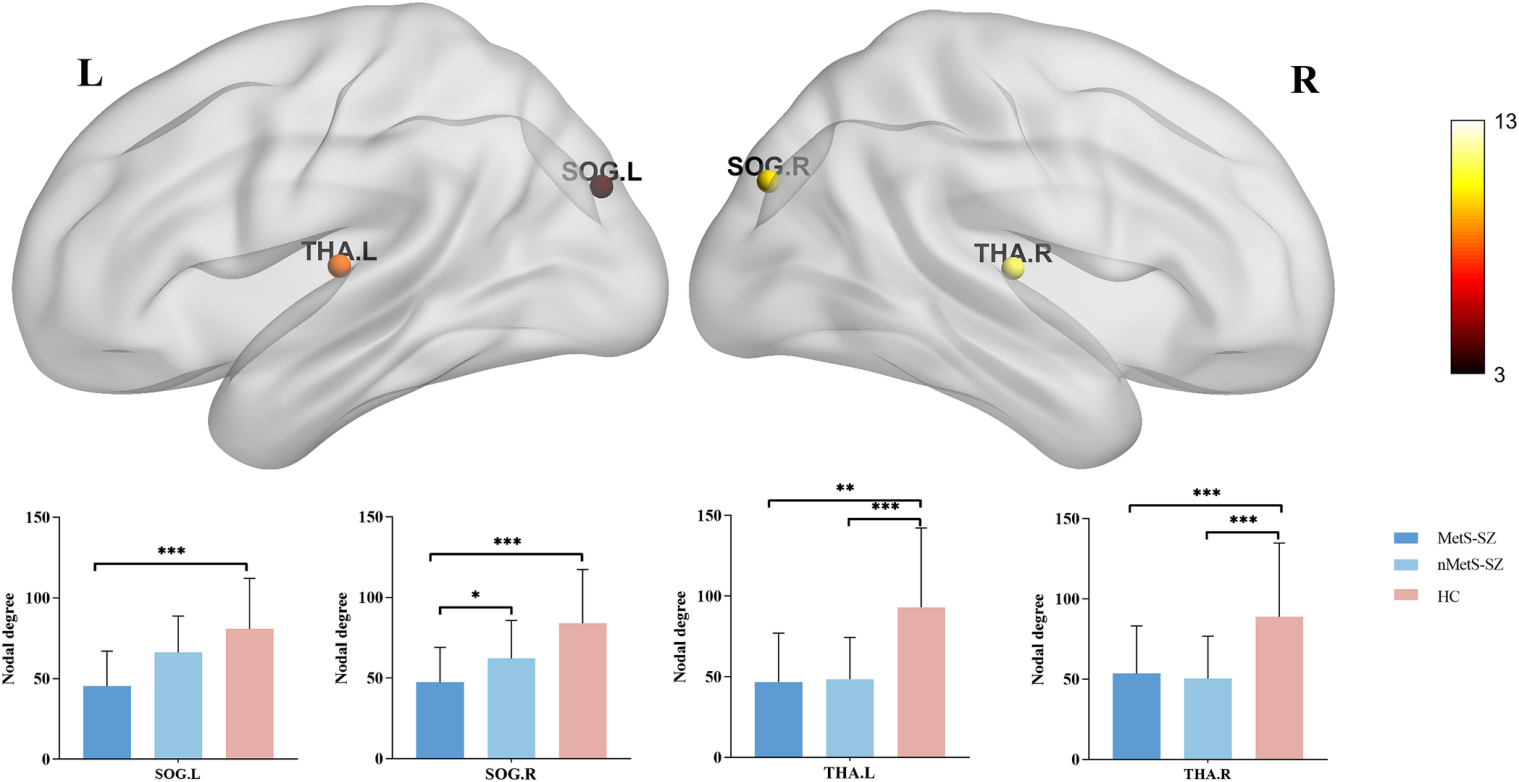
The distribution of brain regions with significant differences in the degree centrality among the groups at *p* < 0.05 (corrected). *Post-hoc* tests showed that MetS and nMetS patients had significant reduced degree centrality in most of these regions compared with controls. SOG, Superior occipital gyrus; THA, Thalamus; L, Left; R, Right; MetS-SZ, Schizophrenia patients with metabolic syndrome; nMetS-SZ, Schizophrenia patients without metabolic syndrome; HC, Healthy controls. *p < 0.05; **p < 0.01; ***p < 0.001.

**Figure 3:**
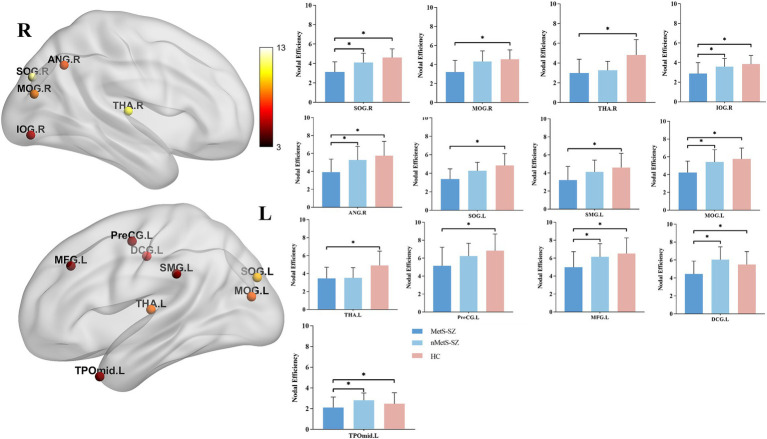
The distribution of brain regions with significant differences in the degree centrality among the groups at *p* < 0.05 (corrected). Post-hoc tests showed that MetS and nMetS patients had significant reduced nodal efficiency in most of these regions compared with controls. SOG, Superior occipital gyrus; MOG, Middle occipital gyrus; IOG, Inferior occipital gyrus; PreCG, Precental gyrus; MFG, Middle frontal gyrus; DCG, Median cingulate and paracingulate gyri; SMG, Supramarginal gyrus; ANG, Angular gyrus, THA, Thalamus; TPOmid, Temporal pole: middle temporal gyrus; L, Left; R, right; MetS-SZ, schizophrenia patients with metabolic syndrome; nMetS-SZ, schizophrenia patients without metabolic syndrome; HC, healthy controls. *p < 0.05; **p < 0.01; ***p < 0.001.

When nodal metrics were evaluated with ANCOVAs, marked in DC were found among groups in the left medial and paracingulate gyrus, bilateral superior occipital gyrus, and bilateral thalamus (corrected *p* < 0.05), as shown in Figure 2. Furthermore, there were marked differences in NE in the left precentral gyrus, left middle frontal gyrus, left medial and paracentral cingulate gyrus, bilateral occipital lobes, right supramarginal gyrus, right angular gyrus, bilateral thalamus, left temporal pole (corrected *p* < 0.05), as shown in Figure 3.

Specifically, relative to HCs, MetS-SZ, and nMetS-SZ patients exhibited significant decreases in DC and NE in the bilateral thalamus. Relative to HCs, reduced DC was seen in the bilateral supraoccipital gyrus of MetS-SZ patients (Figure 2). The NE of patients in the MetS-SZ group was lower than that of HCs in the left precentral gyrus, left middle frontal gyrus, bilateral occipital lobes, right supramarginal gyrus, and right angular gyrus (Figure 3).

Relative to nMetS-SZ patients, those in the MetS-SZ group presented with significant reductions in NE in the left middle frontal gyrus, left medial and paracentral cingulate gyrus, right supraoccipital gyrus, left middle occipital gyrus, right supraoccipital gyrus, right angular gyrus, and left temporal pole.

### 3.3 Associations between network metrics and clinical characteristics

**Figure 4:**
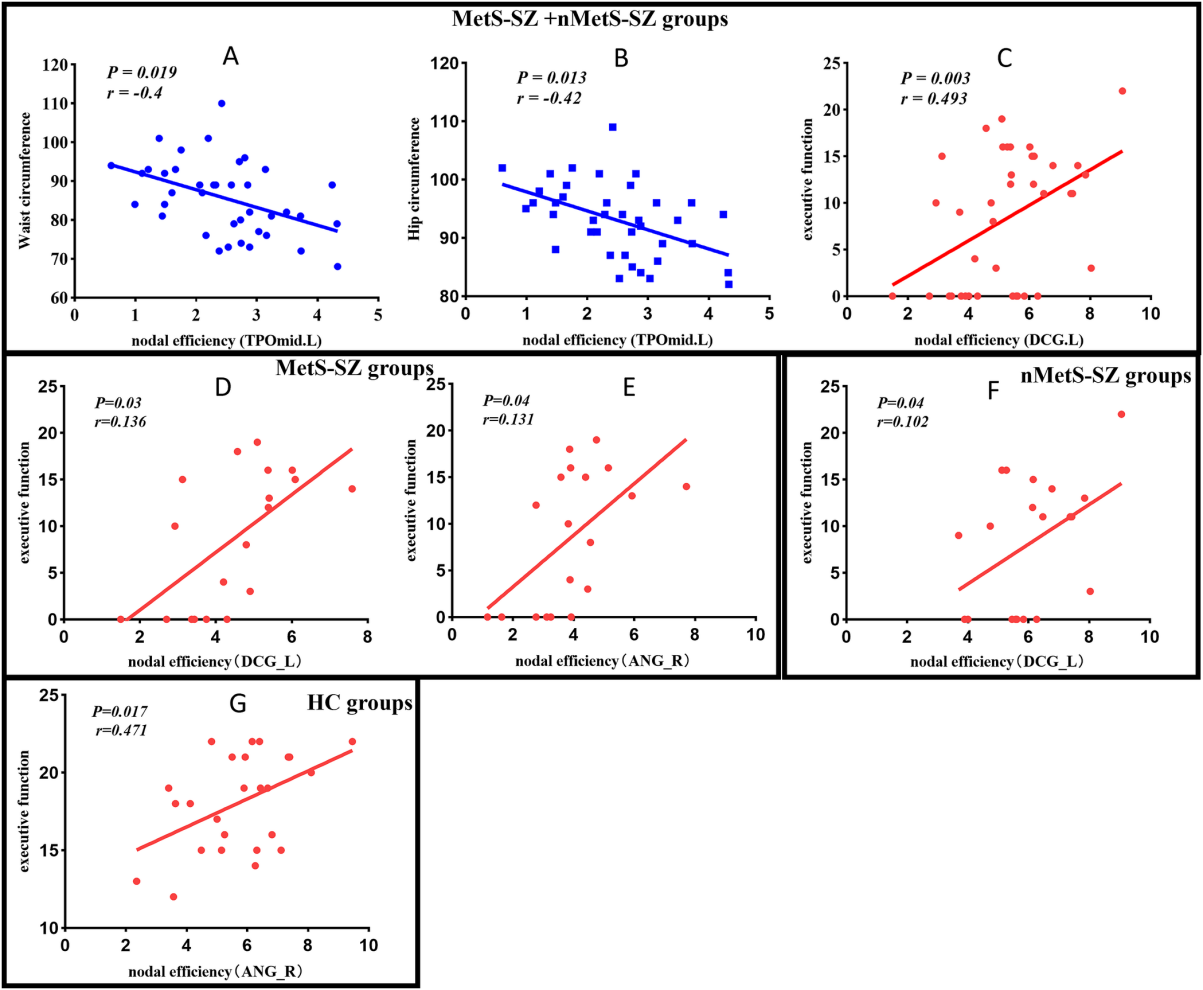
**(A)** Negative correlation between the Waist circumference and nodal efficiency in TPOmid among patients in the MetS-SZ and nMetS-SZ groups. **(B)** Negative correlation between the Hip circumference and nodal efficiency in TPOmid among patients in the MetS-SZ and nMetS-SZ groups. **(C)** Positive correlation between the executive function and nodal efficiency in DCG among patients in the MetS-SZ and nMetS-SZ groups. **(D)** Positive correlation between the executive function and nodal efficiency in DCG among patients in the MetS-SZ groups. **(E)** Positive correlation between the executive function and nodal efficiency in ANG among patients in the MetS-SZ. **(F)** Positive correlation between the executive function and nodal efficiency in DCG among patients in the nMetS-SZ groups. **(G)** Positive correlation between the executive function and nodal efficiency in DCG among patients in the HC groups. MetS-SZ, schizophrenia patients with metabolic syndrome; nMetS-SZ, schizophrenia patients without metabolic syndrome; TPOmid, Temporal pole: middle temporal gyrus; DCG, Median cingulate and paracingulate gyri; ANG, Angular gyri.

In the MetS-SZ group, impairments in executive function were associated with reduced NE values in the right angular gyrus (*p* = 0.04, *r* = 0.131), left medial and paracingulate gyrus (*p* = 0.04, *r* = 0.136). In contrast, higher executive function scores in the nMetS-SZ group were associated with increased NE values in the left medial and paracingulate gyrus (*p* = 0.04, *r* = 0.102). Furthermore, among patients in the MetS-SZ and nMetS-SZ groups, higher waist circumference and hip circumference were found to be potentially related to a decrease in NE in the left temporal pole (*p* = 0.019, *r* = −0.4; *p* = 0.013, *r* = −0.42). The impairment of executive function in these schizophrenia patients was associated with a reduction in NE in the medial and paracrine cingulate gyrus (*p* = 0.03, *r* = 0.493). In HC, higher executive function may be associated with higher NE values in the angular gyrus (*p* = 0.017, *r* = 0.471) (Figure 4). No significant correlations were found between other metabolic indicators, clinical scale scores, and differential brain regions.

## 4 Discussion

The present analysis is the first report exploring changes in white matter network in schizophrenia patients diagnosed with comorbid MetS undergoing risperidone or clozapine treatment. These analyses revealed significant reductions in white matter network DC and NE in both MetS-SZ and nMetS-SZ patients, with the thalamus being particularly profoundly affected. Relative to nMetS-SZ and HC subjects, MetS-SZ patients presented with reductions in both global efficiency and local efficiency. These MetS-SZ patients also exhibited NE values that were reduced more substantially than those for nMetS-SZ patients, with these differences being most pronounced in the prefrontal cortex, angular gyrus, occipital lobe, and temporal pole. White matter network changes in schizophrenia patients groups were associated with cognitive functional impairment and increased waist circumference and hip circumference. This suggests that schizophrenia patients with MetS present with distinctive white matter network alterations, potentially providing a foundation for the development of new targets for clinical development efforts.

Here, MetS-SZ patients presented with reductions in global efficiency and local efficiency relative to nMetS-SZ patients. Global efficiency corresponds to the ability of brain networks to integrate information, whereas local efficiency corresponds to the ability of brain networks to separate information (Watts and Strogatz, 1998; Bassett and Bullmore, 2017). Functional connectivity studies on MetS have shown that MetS is one of the most important factors in disrupting network connections between brains, thereby increasing the risk of cognitive decline in patients (Rashid et al., 2021; Foret et al., 2021). One study indicated that when patients with both MetS and white matter hyperintensities have their global and local efficiency reduced (Zheng et al., 2023), which is consistent with our findings. MetS-SZ patients may thus face a reduction in their ability to process information independently in different brain networks.

The MetS-SZ patients in this study presented with reduced NE in medial prefrontal lobe relative to nMetS-SZ patients which was evident in the middle frontal gyrus and medial and paracingulate gyrus. The medial prefrontal cortex is located in the anterior of the brain and is associated with emotional or intuitive decision-making. This region is closely related to obesity, as functional inhibition in this region contributes to increased appetite, contributing to obesity and thus to MetS development (Lowe et al., 2019). In animal experiments, mice with impaired insulin resistance and dyslipidemia exhibited abnormal malondialdehyde levels in the prefrontal cortex (Veniaminova et al., 2020). One structural MRI study results indicating that MetS-SZ patients present with volume reductions in ventral medial prefrontal reward-related structures (de Nijs et al., 2018). This is consistent with our findings suggesting that medial prefrontal damage may be involved in the pathogenesis and pathophysiology of the MetS. Here, patients in the MetS-SZ group presented with lower angular gyrus NE. The angular gyrus, which is associated with cognitive function and self-regulation, is situated posterior to the lower portion of the parietal lobe (Seghier, 2013). These functions have a direct impact on eating behavior and weight management. MetS has been suggested to be linked to the impairment of angular gyrus white matter microstructural integrity (Alfaro et al., 2016). One white matter study showed that patients with obesity and type 2 diabetes had reduced integrity and white matter volume at the right inferior parietal lobe (van Bloemendaal et al., 2016). This is consistent with our findings, suggesting damaged the angular gyrus leads to reduced decision-making and self-regulation of the brain, and loss of control over the need to eat, leading to obesity and even MetS. MetS onset in post-traumatic stress disorder patients has previously been suggested to be correlated with reductions in the thickness of the temporal and occipital cortices (Wolf et al., 2016). In this study, decreased NE was evident in the occipital region and the temporal pole in MetS-SZ patients. The occipital lobe is closely related to vision, and the present results may thus suggest that schizophrenia patients with MetS face deficient visual information processing, potentially more susceptible to food appearance and seductive (García-García et al., 2015; Zhang et al., 2018). MetS-SZ patients may thus present with more pronounced deficits in terms of information segregation at the junction of the prefrontal lobes, angular gyrus, and temporal pole, offering insight into an abnormality that may be distinctly associated with this subset of patients.

Relative to HC individuals, MetS-SZ and nMetS-SZ patients presented with reduced DC and NE in thalamus. The thalamus is a crucial component of various brain functioning circuits that are impaired in schizophrenia (Byne et al., 2009). Previous research has indicated that individuals with schizophrenia may exhibit structural or functional abnormalities in the thalamus (Wang et al., 2024; Adriano et al., 2010). This finding may indicate that thalamic anomalies are associated with schizophrenia.

In the MetS-SZ group, impaired cognitive function was associated with lower NE in the right angular gyrus, left medial gyrus and cingulate gyrus. Conversely, in the nMetS-SZ group, higher NE in these regions correlated with better executive function. These findings suggest that MetS may impair cognitive function in schizophrenia through altered NE levels in key brain areas, consistent with previous research linking MetS to cognitive dysfunction via white matter changes (Zheng et al., 2023). In healthy controls, higher NE in the angular gyrus was associated with better executive function, indicating its regulatory role in cognitive performance. A possible link between impaired cognitive function and a reduction in the NE of the medial and paracingulate cingulate gyrus among patients with schizophrenia. In depression research, cognitive deficits had also been linked to abnormal functional connectivity in the prefrontal cortex (Zhang et al., 2022). This is similar to our results, suggesting that organized brain networks are vital to maintaining brain function that is intact (Narr and Leaver, 2015). Moreover, in schizophrenia group, a relationship was noted between increases in waist-hip circumference and decreasing temporal pole NE. In a prior report, schizophrenia patients were found to exhibit selective increases in prefrontal and temporal cortical GTP-sensitive 5-HT1A sites (Selvaraj et al., 2014). The antipsychotic drugs in this study work by binding to various 5-HT receptors. Binding to 5-HT receptors in the temporal lobe reduces receptor availability, leading to side effects such as hunger and weight gain (He et al., 2022). Taken together, these findings highlight the critical role of the temporal pole in regulating central obesity. This may be an important mechanism linking changes in waist-hip circumference with brain function, particularly in relation to white matter changes. However, MetS involves multiple metabolic abnormalities, and its effects on the brain may be more complex than a single change in waist circumference. Therefore, in MetS-SZ patients, the relationship between waist-hip circumference and brain network may not be as pronounced as in the overall schizophrenia group.

This study has several limitations. One important limitation is that this study did not employ a longitudinal design. Therefore, we could not account for the potential effects of individual heterogeneity related to psychiatric medication responses, particularly the use of Risperidone and Clozapine. Both of these medications have been associated with metabolic abnormalities (MetS) in previous studies, but their specific impact on the brain structure and function in schizophrenia patients was not directly examined in our study. Given that Risperidone and Clozapine are commonly prescribed in clinical practice and are known to be linked with MetS, their potential influence on our findings cannot be excluded. However, because the study was cross-sectional, and the data available did not allow for a detailed analysis of the independent effects of these medications, it remains unclear whether the observed associations between MetS and brain structure are driven by the medication itself or by the underlying illness. Secondly, this study had a small sample size, and these results may not be more broadly applicable given that all included patients were undergoing treatment with risperidone or clozapine. Finally, one limitation of this study is its inability to distinguish the independent effects of each MetS component. Since the study focused on the overall impact of MetS, it did not investigate the specific effects of individual metabolic factors on the white matter network and cognitive function. Future research should separately evaluate these effects in schizophrenia patients to enhance our understanding of the underlying mechanisms.

In summary, the present results suggest that patients with comorbid schizophrenia and MetS undergoing antipsychotic treatment present with more severe white matter network metrics abnormalities as compared to schizophrenia patients without MetS or healthy individuals. These findings provide a foundation for further studies of the mechanisms within the brain that are linked to comorbid MetS among schizophrenia patients.

## Data Availability

The raw data supporting the conclusions of this article will be made available by the authors, without undue reservation.

## Footnotes

^1^ http://www.fmrib.ox.ac.uk/fsl

^2^ http://www.nitrc.org/projects/panda

^3^ http://www.nitrc.org/projects/gretna/

^4^ https://posit.co/downloads/
